# Pennsylvania Institution for the Instruction of the Blind

**Published:** 1860-11

**Authors:** 


PENNSYLVANIA INSTITUTION FOR THE INSTRUCTION OF THE BLIND.—Dr. Richard J. Dunglison and Dr. S. Weir Mitchell have been appointed physicians to this institution.

